# Evaluating Polylactic Acid and Basalt Fibre Composites as a Potential Bioabsorbable Stent Material

**DOI:** 10.3390/polym17141948

**Published:** 2025-07-16

**Authors:** Seán Mulkerins, Guangming Yan, Declan Mary Colbert, Declan M. Devine, Patrick Doran, Shane Connolly, Noel Gately

**Affiliations:** 1 PRISM Research Institute, Technological University of the Shannon, University Road, N37HD68 Athlone, Ireland; guangming.yan@tus.ie (G.Y.); declan.colbert@tus.ie (D.M.C.); 2 Department of Polymer, Mechanical & Design, Technological University of the Shannon, University Road, N37HD68 Athlone, Ireland; patrick.doran@tus.ie; 3 Applied Polymer Technology Gateway, Technological University of the Shannon, University Road, N37HD68 Athlone, Ireland; shane.connolly@tus.ie; 4 Technology Transfer Office, Technological University of the Shannon, University Road, N37HD68 Athlone, Ireland; noel.gately@tus.ie

**Keywords:** bioabsorbable polymer stents, polylactic acid, basalt fibre, twin-screw extrusion

## Abstract

Bioabsorbable polymer stents (BPSs) were developed to address the long-term clinical drawbacks associated with permanent metallic stents by gradually dissolving over time before these drawbacks have time to develop. However, the polymers used in BPSs, such as polylactic acid (PLA), have lower mechanical properties than metals, often requiring larger struts to provide the necessary structural support. These larger struts have been linked to delayed endothelialisation and an increased risk of stent thrombosis. To address this limitation, this study investigated the incorporation of high-strength basalt fibres into PLA to enhance its mechanical performance, with an emphasis on optimising the processing conditions to achieve notable improvements at minimal fibre loadings. In this regard, PLA/basalt fibre composites were prepared via twin-screw extrusion at screw speeds of 50, 200, and 350 RPM. The effects were assessed through ash content testing, tensile testing, SEM, and rheometry. The results showed that lower screw speeds achieved adequate fibre dispersion while minimising the molecular weight reduction, leading to the most substantial improvement in the mechanical properties. To examine whether a second extrusion run could enhance the fibre dispersion, improving the composite’s uniformity and, therefore, mechanical enhancement, all the batches underwent a second extrusion run. This run improved the dispersion, leading to increased strength and an increased modulus; however, it also reduced the fibre–matrix adhesion and resulted in a notable reduction in the molecular weight. The highest mechanical performance was observed at 10% fibre loading and 50 RPM following a second extrusion run, with the tensile strength increasing by 20.23% and the modulus by 27.52%. This study demonstrates that the processing conditions can influence the fibres’ effectiveness, impacting dispersion, adhesion, and molecular weight retention, all of which affect this composite’s mechanical performance.

## 1. Introduction

Coronary artery disease (CAD) is a major global health issue, with 7 million deaths occurring worldwide annually [1]. In the treatment of CAD, cardiovascular stents have been a significant development, providing a minimally invasive solution for the long-term support of narrowed or weakened arteries. There are primarily two types of stents used in CAD treatment—permanent metallic stents and bioabsorbable stents.

Metallic stents currently dominate the global stent market due to their excellent mechanical properties, namely their high strength, Young’s modulus, and flexibility, which have enabled the development of stents with exceptionally low strut thicknesses [2,3]. This feature reduces the risk of target lesion revascularisation and stent thrombosis [4] and improves the deliverability to small or complex vasculature [5]. However, the permanent presence of a stent within an artery can pose several clinical drawbacks, including in-stent restenosis [6], late and very late stent thrombosis, neo-atherosclerosis, impaired vasomotion due to the permanent caging of the artery [7], delayed endothelialisation [8], and instability in growing vessels for paediatric patients [9]. These drawbacks represent a significant disadvantage of using metallic stents.

In contrast, bioabsorbable polymer stents (BPSs) offer the potential to revolutionise the treatment of CAD and significantly improve the patient outcomes. By supporting the artery throughout the remodelling period and then being fully absorbed by the body, BPSs could theoretically eliminate the aforementioned clinical drawbacks associated with metallic stents. Nevertheless, despite their potential benefits, BPSs often have inferior mechanical properties compared to metals, typically requiring oversized struts as a form of compensation [10]. However, oversized struts have been directly linked with delays to the endothelialisation process, subsequently increasing the risk of stent thrombosis [11]. While research and development in this field continues, the current mechanical limitations of bioabsorbable stents must be addressed before they can be regarded as a viable alternative to metallic stent materials [3].

Polylactic acid (PLA) has been extensively investigated as a material for bioabsorbable stent use. It has several attractive features, including a predictable degradation profile; good biocompatibility, thermal stability, and mechanical properties; and excellent mechanical integrity [12,13]. Although PLA’s mechanical properties are considered advantageous among the established bioabsorbable polymers, they remain inferior to those of metallic stents. While significant developments have been made in enhancing PLA’s inherent brittleness using secondary processing methods such as biaxial expansion and annealing [14,15], the improvements to its tensile strength and Young’s modulus have been less pronounced. One method that has been shown to address these limitations is the addition of reinforcing fibres, such as basalt fibre.

Basalt fibre is a natural, inorganic fibre made from basalt rock and is increasingly being used as a reinforcing fibre for polymer materials. It has several attractive properties, including its excellent tensile strength and modulus, directly compensating for the inherent limitations of PLA [16]. Consequently, incorporating basalt fibre into PLA could allow for the adoption of a thinner strut profile when compared to using PLA alone. In this regard, several studies have demonstrated significant improvements in this area. For example, Czigány et al. found that incorporating 40% basalt fibre into PLA led to a 91.4% increase in the tensile strength (64.3 MPa to 123.5 MPa) and 179% increase in Young’s modulus (2.97 GPa to 8.31 GPa) [17]. Additionally, Han et al. reported that the addition of 50% basalt fibre content improved the tensile strength of PLA from 58.3 MPa to 110.2 MPa (89.02%) [18].

In addition to mechanical improvement, basalt fibre has also demonstrated promising biocompatibility. While further research is needed to assess its suitability in vascular applications, early research suggests it does not elicit an adverse biological response. For example, long-term in vivo studies have assessed the potential carcinogenicity of basalt fibre compared to that of asbestos exposure. Kogan et al. [19] and McConnell et al. [20] found that while asbestos exposure led to significant morbidity and mortality in rats, basalt fibre showed no signs of carcinogenic activity, even at exposure levels ten times higher than those used for asbestos. In later research, basalt fibre was incorporated into PLA to fabricate a composite for hard tissue repair. The study evaluated its cytocompatibility by growing osteoblasts on both flat films and three-dimensional scaffolds, finding that the osteoblasts grew equally well on PLA and PLA/BF composites. This suggests that the incorporation of basalt fibre does not negatively impact cell growth, indicating its potential to also support endothelial cell growth [21].

Consequently, these studies suggest that basalt fibre is a viable reinforcement material for use in a bioabsorbable stent, offering both biocompatibility and mechanical benefits. However, while the effectiveness of basalt fibre in enhancing PLA’s mechanical properties has been demonstrated, prior studies achieved this primarily by increasing the fibre loading. This inherently reduces the proportion of the PLA matrix, which would likely lead to a faster overall degradation rate compared to that of a purely PLA-based stent, potentially compromising the structural integrity before complete endothelialisation. As a result, premature degradation could lead to the early detachment of fibres, which could subsequently enter the bloodstream and thereby significantly increase the risk of an arterial blockage. Therefore, an alternative approach is to optimise the processing parameters to maximise the reinforcing effect of basalt fibres at lower loadings, thereby preserving a higher proportion of the PLA matrix and thus mitigating the risk of premature degradation. However, to our knowledge, there is a lack of published research investigating whether process optimisation can enhance the effectiveness of basalt fibres in PLA composites.

Consequently, this study aimed to investigate the influence of process conditions—specifically the screw speed and a secondary extrusion—on the mechanical properties of PLA/basalt fibre composites at low loadings. The screw speed was chosen due to its established role in enhancing the fibre dispersion and mechanical strength in PLA-based composites [22]. A secondary extrusion run was introduced to address an observed tendency for basalt fibres to separate from PLA during feeding, which was expected to negatively impact the mixing and subsequent dispersion. By reprocessing the material, the second extrusion run was intended to promote additional mixing and improve the distribution of the fibres throughout the matrix. Therefore, by examining these factors, this study aimed to determine whether the selected processing parameters can meaningfully contribute to maximising the mechanical performance of basalt fibre in a composite material.

## 2. Materials and Methods

### 2.1. Materials

Polylactic acid (PLA) (Luminy^®^ LX175) was obtained from Total Corbion (Gorinchem, The Netherlands) in pellet form. The material had a density of 1.24 g/cm^3^ and a melt flow index of 8 g/10 min (210 °C/2.16 kg). It was a semi-crystalline hygroscopic material with a glass transition temperature (Tg) of 60 °C and a melt temperature (Tm) of 155 °C. Its mechanical properties included a Young’s modulus of 3500 MPa, a tensile strength of 50 MPa, and an elongation at break of <5%. Chopped strands of basalt fibre treated with a silane sizing were obtained from Kamenny Vek Ltd. (Moscow, Russia). The fibres had a nominal cut length of 12.7 mm and an approximate monofilament diameter of 13 µm.

### 2.2. Preparation of PLA/BF Composites

Prior to processing, all the materials were dried for 6 h in a convection oven set to 85 °C to reduce the moisture content to below 0.01%, as confirmed using an OHAUS MB90 moisture analyser (OHAUS Corporation, Parsippany, NJ, USA). The materials were then weighed and subsequently transferred into sealed polyethylene bags, each containing 1 kg in total of the PLA/basalt fibre mixture. The contents of each bag were tumble-mixed for 5 min to ensure a homogeneous distribution of the fibres within the PLA. The blend compositions for each batch are shown in Table 1 below.

### 2.3. Hot-Melt Extrusion of Composites

A co-rotating twin-screw extruder (EUR.EX.MA Tradate, Italy) equipped with a 2 mm filament die was used to produce filaments from both virgin PLA and the PLA/basalt fibre composites. The extruder featured eight heating zones from the hopper to the die, set at 180 °C, 180 °C, 185 °C, 185 °C, 185 °C, 185 °C, 190 °C, and 190 °C, respectively, in accordance with the manufacturer’s processing recommendations. The screw speeds were varied at 50, 200, and 350 RPM to investigate how the shear rate influences the fibre dispersion and, in turn, the mechanical properties. The extruder had a screw diameter of 21.7 mm and a length-to-diameter (L/D) ratio of 40. Upon exiting the die, the filament extrudate was cooled using an air-cooled haul-off system and subsequently pelletised into approximately 2 mm pellets.

To investigate the effect of a secondary extrusion run on the fibre dispersion and batch uniformity, each 1 kg batch (see Table 1 for composition) was divided in half following the first extrusion. One portion (0.5 kg) was designated as ‘run 1’ (single extrusion), while the other underwent a second extrusion under identical conditions. This allowed for a direct comparison of the fibre dispersion and mechanical properties between single- and double-extruded samples.

### 2.4. Injection Moulding

The injection moulding of Type V tensile test bars (per ASTM D638 [23] was performed using a fully hydraulic Babyplast micro-injection moulding machine. The samples were moulded under a temperature profile ranging from 180 °C at the hopper to 190 °C at the nozzle, with a mould temperature of 30 °C and a cooling time of 30 s. The injection pressure, speed, holding time, and cooling time were set to 60 bar, 55%, 3.5 s, and 40 s, respectively. Following moulding, the samples were immediately sealed in polyethylene bags to prevent moisture uptake.

### 2.5. Content Uniformity (Ash Content Analysis)

The ash content of each composite was determined using a Carbolite Gero furnace to assess the variations in the fibre content between individual samples, providing an indirect measure of the batch uniformity and fibre dispersion. The testing was performed in accordance with ASTM D5630 [24]. Three samples from each batch were first weighed on an analytical scale, with each sample having a mass of between 4 and 6 mg. The samples were placed in pre-weighed crucibles and heated to 800 °C for 30 min to ensure the complete evaporation of the PLA. After heating, the samples were cooled to an ambient temperature and subsequently reweighed to determine the amount of remaining ash residue, corresponding to the actual fibre content. The basalt fibre content was calculated as a percentage of the initial sample weight using Equation (1):

(1) $$Actual\;Basalt\;Fibre\;(\%)=\frac{\left(W_{3}-W_{1}\right)}{\left(W_{2}-W_{1}\right)}\times 100$$

where

*W*_1_ = the mass of the sample crucible (g);

*W*_2_ = the mass of the sample crucible (g) and the mass of the sample (g);

*W*_3_ = the mass of the sample crucible (g) and the ash residue (g).

### 2.6. Fracture Surface Morphology

The fracture surface morphology was observed using scanning electron microscopy (SEM). The fracture site of the samples used for testing was examined following tensile testing. The specimens were affixed to aluminium sample holders and then coated using an Agar Gold Sputter Coating machine prior to analysis. SEM analysis was conducted with a TESCAN MIRA SEM, operated at 20 kV with magnifications of 50, 500, and 1000×, utilising the backscattered electron (BSE) mode. This mode employed high-energy electrons to capture detailed images, illustrating the arrangement of different constituent elements within the sample. SEM analysis was performed on representative samples to assess the effects of the basalt fibre loading, screw speed, and extrusion run conditions, as outlined in Table 2 below.

### 2.7. Rheological Analysis

**Figure 1:**
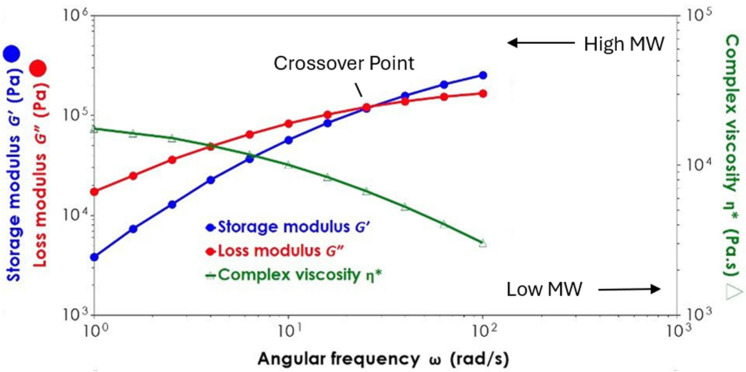
Crossover point of storage modulus (G′) and loss modulus (G″) as indicator of molecular weight. Shift to higher frequencies signifies molecular weight reduction. Adapted from [26].

Rheological measurements were conducted using a TA Instruments Discovery Hybrid Rheometer in the oscillatory mode. The testing was performed using a parallel plate configuration (25 mm plate diameter, 1000 μm gap) at a temperature of 190 °C, consistent with that used during the extrusion process to ensure comparable conditions. First, an amplitude sweep was carried out to determine the linear viscoelastic region (LVR). A frequency sweep was then conducted within this region, ranging from 0.1 to 100 Hz (approximately 0.63 to 628 rad/s) at a constant strain amplitude of 1%, to identify the crossover point of the storage modulus (G′) and loss modulus (G″). This crossover point was subsequently used as an indirect indicator of the molecular weight, as higher-molecular-weight materials tend to exhibit a crossover at lower frequencies [25,26,27,28]. Accordingly, it served as a comparative measure to assess the molecular weight trends across samples processed under different conditions, with a shift to higher crossover frequencies indicating a molecular weight reduction, as shown in Figure 1.

To further evaluate the molecular weight changes, the complex viscosity (η∗) was analysed as a function of the angular frequency (ω). This was then correlated with the steady-shear viscosity (η) as a function of the shear rate (γ˙) using the Cox–Merz rule [29]:

η∗(ω) = η(γ˙) at ω = γ(2)

The zero-shear viscosity (η_0_) was subsequently estimated by fitting the steady-shear viscosity data to the Carreau–Yasuda model [30]:

η(γ˙) = η_0_ [1 + (λγ˙)^a^]^(*n*−1)/*a*^ (3)

where (λ) is the relaxation time, (n) is the power-law index, and (a) is a parameter describing the transition region between the Newtonian plateau and the power-law region. For PLA, the zero-shear viscosity (η_0_) has been shown to scale with the weight-average molecular weight (M_w_) according to the relationship η_0_ ≈ M_w_^3.4^, making it a reliable indicator for assessing molecular degradation [31].

### 2.8. Mechanical Analysis

#### Tensile Testing

The tensile properties of the samples were evaluated using a LLOYD Instruments (Bognor Regis, United Kingdom) universal tensile testing machine equipped with a 2.5 kN load cell, using Type V tensile bars. All the tests were performed under ambient conditions with a constant extension rate of 1 mm/min and a gauge length of 7.62 mm, per ASTM D638-14 [23].

## 3. Results and Discussion

### 3.1. Content Uniformity

Ash content analysis was conducted to quantify the actual basalt fibre content in each sample following each extrusion run. As described in Section 2.3, the extrusion run 2 samples (samples that had undergone a secondary extrusion run) were processed using the same batch as run 1, with no additional fibres introduced. Therefore, the differences observed between the runs can be attributed primarily to variations in the fibre dispersion rather than differences in the total fibre content. The results, including the standard deviation (σ), are summarised in Table 3.

The first extrusion run demonstrated the largest deviations across all the fibre loadings from the target basalt fibre loading at the lowest screw speed (50 RPM), with measured values of 4.10% vs. 5%, 6.48% vs. 7.5%, and 8.71% vs. 10%. To our knowledge, no direct study has quantified the fibre content using the ash content method across varying screw speeds. Therefore, a possible explanation for this trend was drawn from a study on polystyrene/cellulose fibre composites. This study suggested that at low shear rates, reduced screw speeds may be insufficient to break up fibre agglomerates, leading to an uneven distribution [32]. This indicates that the low shear at 50 RPM was less effective at effectively breaking apart fibre clusters, resulting in greater variability and deviations from the target fibre content.

Increasing the screw speed to 200 RPM improved the fibre dispersion, bringing the fibre loadings closer to the target values (4.36% vs. 5%, 7.08% vs. 7.5%, and 10.12% vs. 10%), likely due to more effective fibre deagglomeration. However, increasing the speed to 350 RPM resulted in only marginal improvements, suggesting a threshold beyond which additional shear no longer significantly enhanced the dispersion. Notably, when examining the effect of the fibre loading independent of the screw speed, batches with 10% fibre loading processed at 200 and 350 RPM showed a lower deviation from the target fibre content than those with 5 and 7.5% loadings under the same conditions. This trend likely reflects the higher fibre concentration at 10%, which promoted a more uniform distribution throughout the matrix, minimising the PLA-rich regions within the composite.

**Figure 2:**
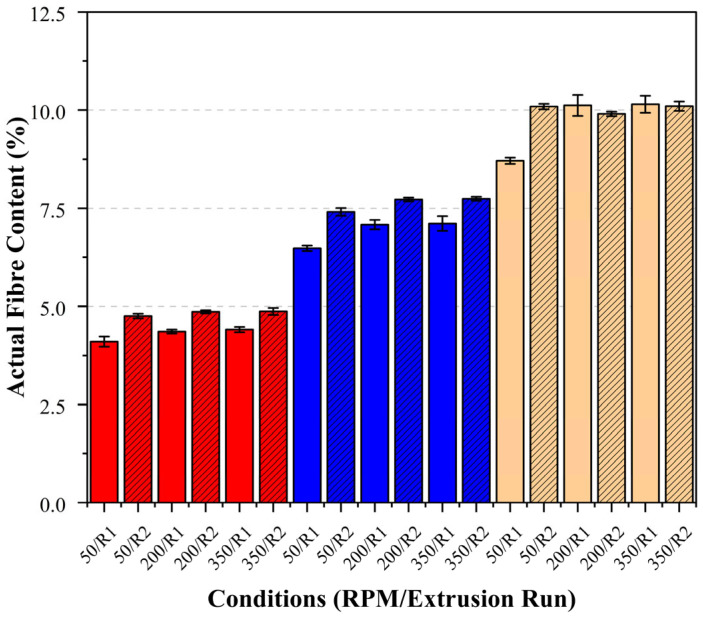
Actual fibre content (%) measured via ash content analysis for the PLA/basalt fibre composites under different fibre loadings and processing conditions. Fibre loadings of 5%, 7.5%, and 10% are represented in red, blue, and beige, respectively. The grey dashed lines represent the target fibre loadings for each condition. Solid bars indicate a single extrusion run, while hatched bars indicate a second extrusion run.

Following the second extrusion run, all the samples exhibited fibre content measurements more consistent with the target values across all the screw speeds. This effect was attributed to the cumulative influence of the repeated processing steps, where the initial extrusion and pelletising, combined with additional mixing, promoted a more uniform fibre dispersion within the PLA matrix. The actual fibre content (%) measured via ash content analysis for the PLA/basalt fibre composites, comparing the effects of the fibre loadings and screw speeds for each extrusion run, is presented in Figure 2 below.

### 3.2. Fracture Surface Morphology

As outlined in Section 2.6, SEM analysis was conducted on selected representative samples to evaluate the effects of the fibre loading, screw speed, and secondary extrusion. Images were captured at 50, 500, and 1000× magnifications, with the corresponding results analysed in the subsequent sections.

#### 3.2.1. Effect of Fibre Loading at Constant Screw Speed

**Figure 3:**
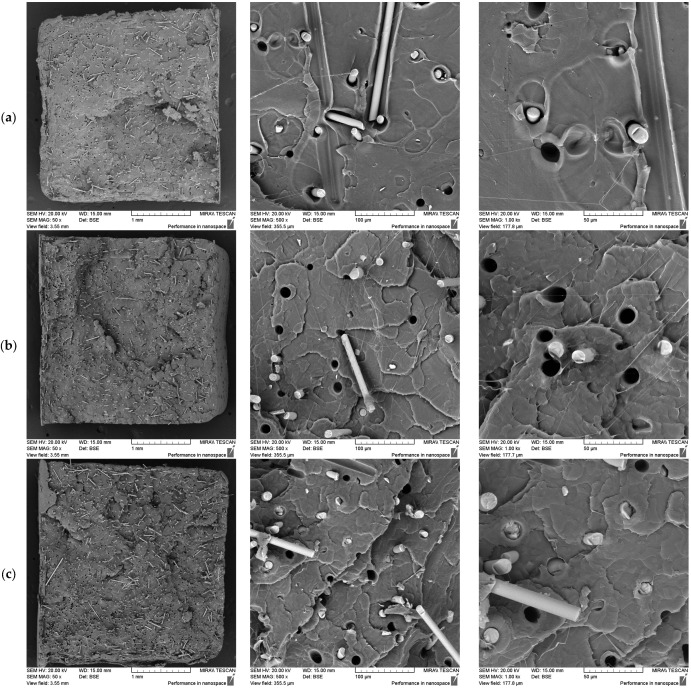
SEM images of fractured tensile samples at 5% (**a**), 7.5% (**b**), and 10% (**c**) basalt fibre loadings, processed at 200 RPM, showing fibre dispersion and fibre–matrix adhesion at increasing magnifications.

The influence of the fibre loading on the composites’ properties was assessed using PLA/basalt fibre composites containing 5%, 7.5%, and 10% fibre, all processed at a fixed screw speed of 200 RPM. The corresponding results are presented in Figure 3.

The fibre dispersion was generally uniform across all the loadings, with no evident signs of agglomeration. However, a notable feature across all the samples was the presence of fibre pull-out, which provides insights into the fibre–matrix adhesion. This was reflected in the uniform voids left behind, indicating weak fibre–matrix bonding, as the fibres were removed cleanly without significant deformation or cracking, suggesting minimal resistance from the surrounding material. The poor adhesion observed can be attributed to the inherently low surface energy of basalt fibres as a result of their high chemical inertness [33,34]. Furthermore, despite the use of silane sizing, which has been shown to enhance bonding [35,36], the lack of matrix restriction observed in this study suggests that it was not effective under the conditions tested. The extent of the pull-out was consistent across the loadings, suggesting that the loading itself does not affect adhesion. Similarly, Han et al. observed significant fibre pull-out due to weak adhesion in silane-treated PLA/basalt fibre composites at a 10% loading [37]. Interestingly, their study found that the adhesion improved with an increasing fibre loading, attributing this to a higher packing density, which provided greater matrix constraint at higher fibre concentrations. This suggests that the fibre loadings used in this study may be insufficient to achieve adequate adhesion with silane treatment alone. Therefore, either higher fibre loadings or alternative adhesion strategies are necessary to improve the bonding at lower concentrations.

#### 3.2.2. The Effect of the Screw Speed at a Constant Fibre Loading

**Figure 4:**
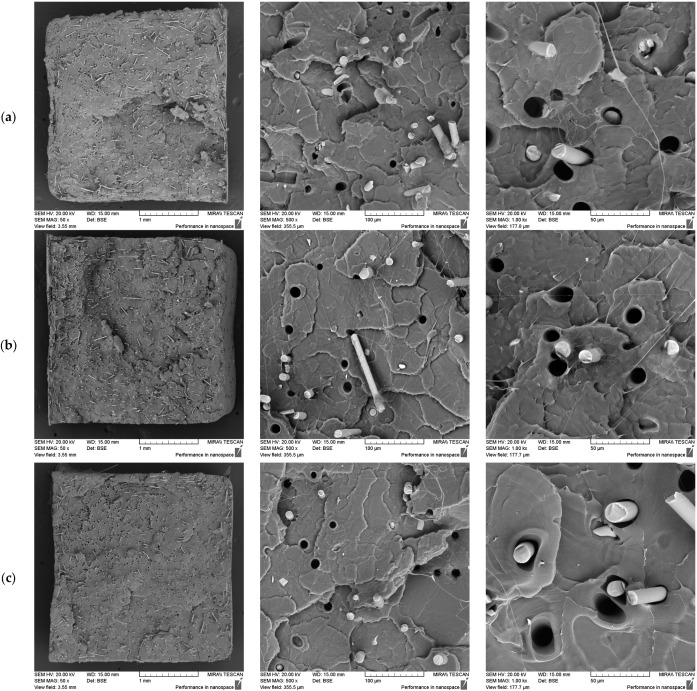
SEM images of fractured tensile samples at 7.5% basalt fibre loading, processed at 50 RPM (**a**), 200 RPM (**b**), and 350 RPM (**c**), showing effect of screw speed on fibre dispersion and fibre–matrix adhesion at increasing magnifications.

The effect of the screw speed was examined using 7.5% basalt fibre composites processed at 50, 200, and 350 RPM, with the results shown in Figure 4 below.

**Figure 5:**
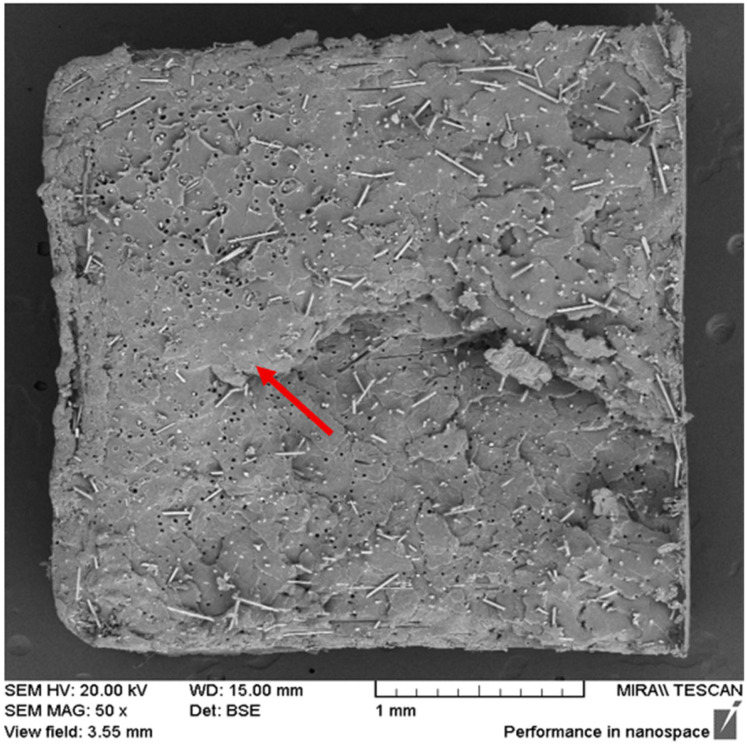
SEM image at 50× magnification of the 7.5% basalt fibre composite processed at 50 RPM. The red arrow highlights a fibre-free region, indicating a less effective fibre distribution compared to that at higher screw speeds.

The fibre dispersion appeared to be uniform across all the conditions; however, the 50 RPM sample exhibited a large fibre-free region at 50× magnification (Figure 5), suggesting less effective dispersion. At 200 and 350 RPM, the fibres appeared to be more evenly distributed, suggesting that higher shear rates improve dispersion.

In regard to the fibre orientation—which is known to influence mechanical properties, with the strength typically enhanced in the direction of orientation [38,39]—distinct differences were observed across the screw speeds. Notably, since the samples underwent a further injection moulding process after extrusion, the final fibre orientation was not solely dictated by the extruder and must be considered accordingly. Nevertheless, given that the Babyplast system uses a hydraulic piston rather than a rotating screw, this likely helped to preserve more of the extrusion-induced orientation, which explains why a clear difference in the fibre alignment was observed across the screw speed variations. For example, at 50 RPM, the fibres were randomly distributed, with this effect most clearly observed in the 50× image, suggesting no preferred reinforcement direction. At 200 RPM, the fibres exhibited a more defined circular flow pattern, suggesting alignment in multiple directions, which may have led to more balanced mechanical properties in both the axial and circumferential directions. At 350 RPM, most fibres were aligned with the tensile direction, suggesting maximum reinforcement along this axis; however, this may also have resulted in reduced strength in other directions.

#### 3.2.3. Effect of Secondary Extrusion at Constant Screw Speed

**Figure 6:**
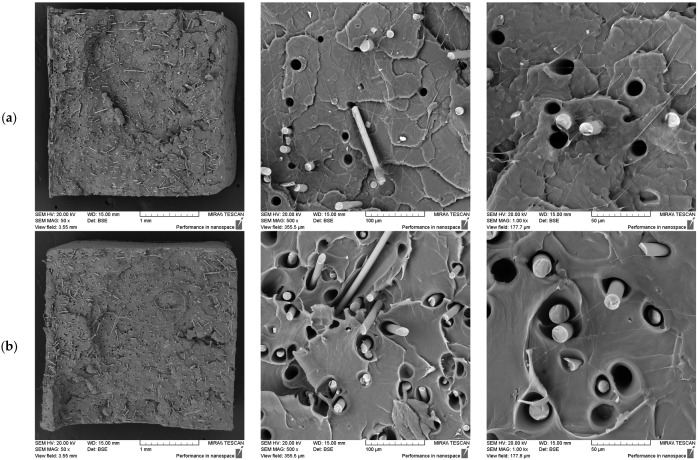
SEM images comparing the effects of a single extrusion run (**a**) and a second extrusion run (**b**) on 7.5% basalt fibre composites processed at 200 RPM.

The influence of a second extrusion run was evaluated by comparing single- and double-extruded samples at a 7.5% fibre loading, processed at a constant 200 RPM. The results are presented in Figure 6 below.

**Figure 7:**
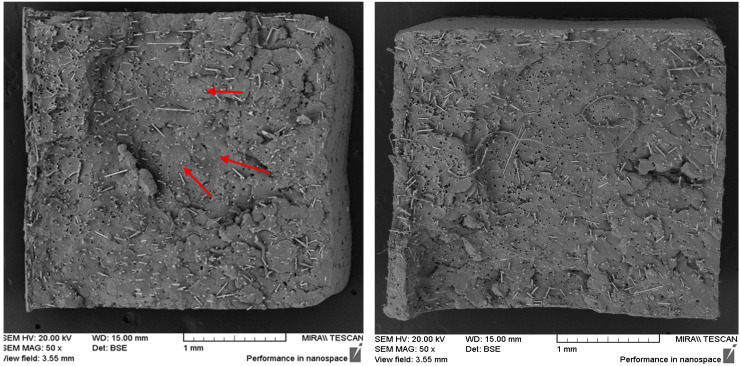
SEM images comparing the fibre dispersion in 7.5% basalt fibre composites processed at 200 RPM after a single extrusion run (**left**) and a second extrusion run (**right**). Red arrows in the single extrusion run highlight large fibre-free regions, indicative of poor dispersion. The composites subjected to a second extrusion run showed improved fibre dispersion with fewer fibre-free regions.

The fibre dispersion was more uniform after the second extrusion run, with fewer fibre-free regions compared to the single-extrusion sample. In contrast, the single-extruded samples exhibited greater variability, particularly by showing larger fibre-free regions near the centre of the sample (Figure 7). This trend was consistent with the ash content analysis, which also indicated improved fibre dispersion following the second extrusion run.

Furthermore, with the additional extrusion cycle, the fibre alignment shifted from a random distribution to being primarily along the tensile axis, as was most evident in the 50× images (see Figure 7). This shift was likely due to flow-induced alignment during extrusion, which became more pronounced with repeated processing. Additionally, the second extrusion run may have facilitated the further breaking up of fibre clusters, allowing individual fibres to better align with the flow direction. However, in contrast, the fibre–matrix adhesion was notably reduced after the second extrusion run, as evidenced by larger and more well-defined fibre pull-out voids. This suggests weaker interfacial bonding, possibly due to the degradation of the PLA matrix or changes to the fibre surface treatment during reprocessing.

### 3.3. Rheological Analysis

Rheological analysis was conducted to evaluate the potential changes in the molecular weight of PLA post-processing. A summary of the results and their standard deviations in presented in Table 4 below.

**Figure 8:**
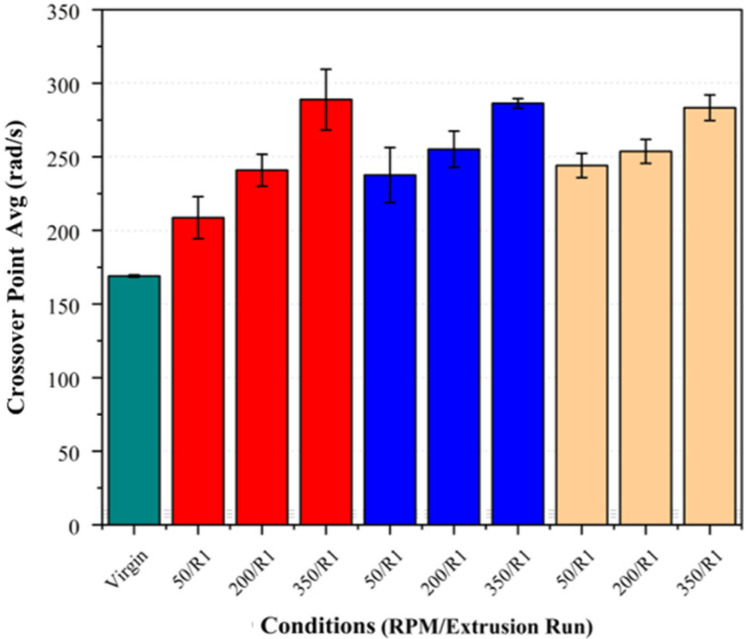
Crossover frequency (G′ = G″) of PLA/basalt fibre composites at varying screw speeds (50, 200, and 350 RPM) and after different extrusion runs (R1, R2). Virgin PLA is shown in green, 5% fibre loading in red, 7.5% in blue, and 10% in beige.

**Figure 9:**
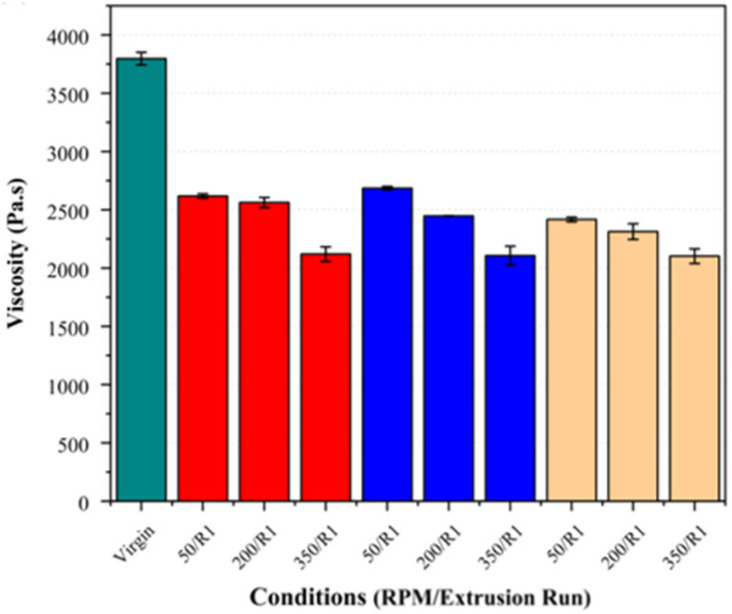
Zero-shear viscosity of PLA/basalt fibre composites at varying screw speeds (50, 200, and 350 RPM) and a single extrusion run Bar colours correspond to fibre loadings as defined in Figure 8.

Increasing the screw speed resulted in notable reductions in the zero-shear viscosity and a shift in the crossover point (G′ = G″) to higher frequencies (see Figure 8 and Figure 9). This effect was observed across all the fibre loadings, with the largest reduction in the viscosity and highest crossover frequency observed at 350 RPM, suggesting that the increased shear at higher screw speeds may contribute to polymer chain scission, leading to a molecular weight reduction.

Interestingly, this observed reduction in the zero-shear viscosity at higher screw speeds, indicating chain scission, contrasts with the existing literature on PLA, which suggests that molecular weight reductions are primarily influenced by the residence time at lower speeds rather than the shear [40,41]. While no direct studies examining the effects of the shear on PLA/basalt fibre composites at varying screw speeds are available, insights can be inferred from the primary degradation mechanisms of PLA.

PLA degradation during melt processing is primarily influenced by the moisture, temperature, residence time, and shear stress [42]. Since these parameters remained consistent across all the samples, the molecular weight degradation inferred in this study appears to have been driven by the presence of the basalt fibres themselves, possibly through a fibre–polymer interaction, thereby causing local shear and subsequent chain scission. To the best of our knowledge, no studies on PLA/basalt fibre composites have specifically examined molecular weight degradation resulting from fibre addition. However, a prior study investigating PLA/basalt fibre composites reported significant increases in the melt flow index (MFI) with an increasing fibre content [43]. The authors attributed this behaviour to higher shear stresses induced by fibre incorporation during processing, leading to polymer chain scission. Since increases in the MFI are directly associated with molecular weight reductions [41], these findings support the trends observed in this study.

**Figure 10:**
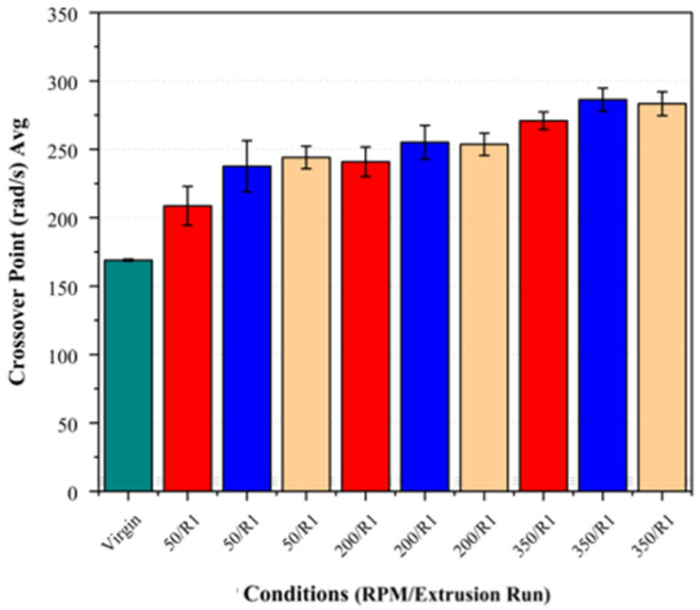
Comparison of crossover frequency (G′ = G″) across different basalt fibre loadings (5%, 7.5%, and 10%) to assess effect of fibre content. Virgin PLA is shown in green, 5% fibre loading in red, 7.5% in blue, and 10% in beige.

**Figure 11:**
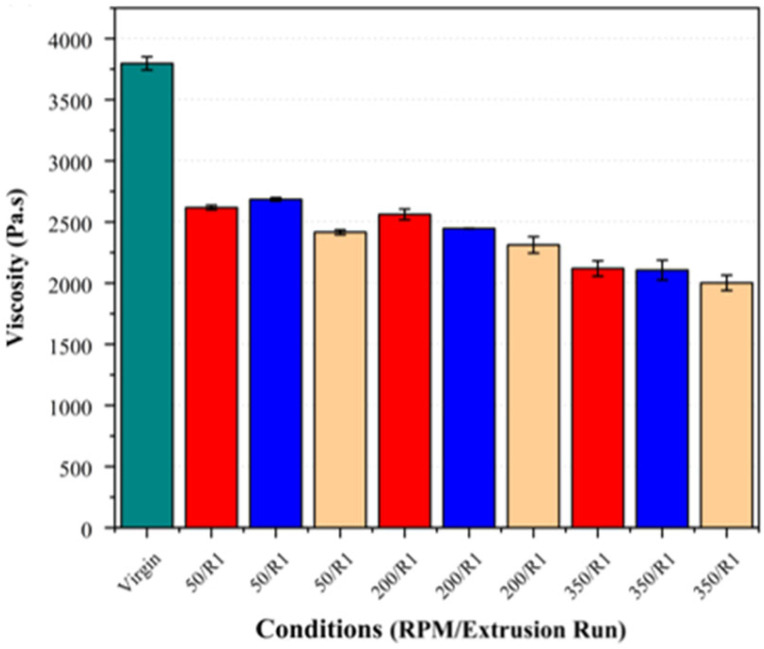
Comparison of zero-shear viscosity across different basalt fibre loadings (5%, 7.5%, and 10%) to assess effect of fibre content. Bar colours correspond to fibre loadings as defined in Figure 10.

The effect of the fibre loading was also examined independently at each screw speed to assess its influence on the crossover point and zero-shear viscosity. As shown in Figure 10 and Figure 11, there was no clear trend indicating that the fibre loading significantly influenced these parameters. While minor variations were present, they largely fell within the standard deviation, suggesting that the fibre loading did not exhibit a measurable impact on these rheological properties, indicating no additional chain scission attributable to the fibre loading.

**Figure 12:**
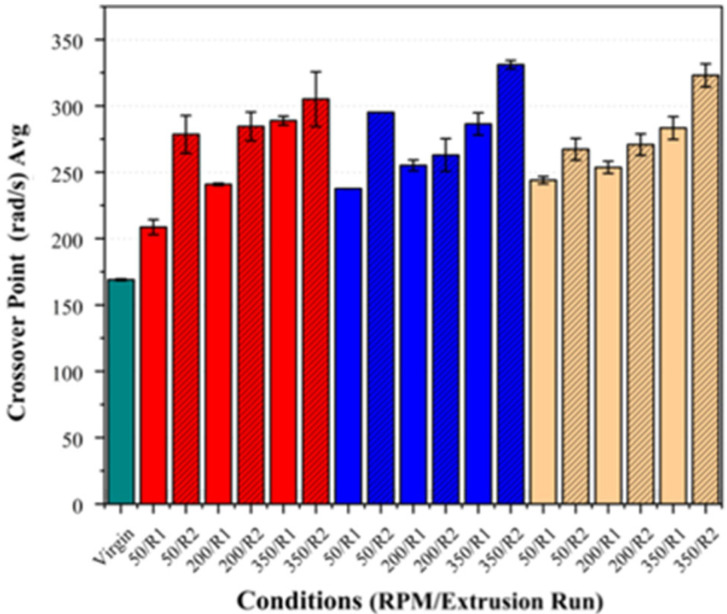
Crossover frequency (G’ = G”) of PLA/basalt fibre composites at varying screw speeds (50, 200, and 350 RPM) and after different extrusion runs (R1, R2). Virgin PLA is shown in green, 5% fibre loading in red, 7.5% in blue, and 10% in beige. Hatched bars represent samples processed using second extrusion run (R2).

**Figure 13:**
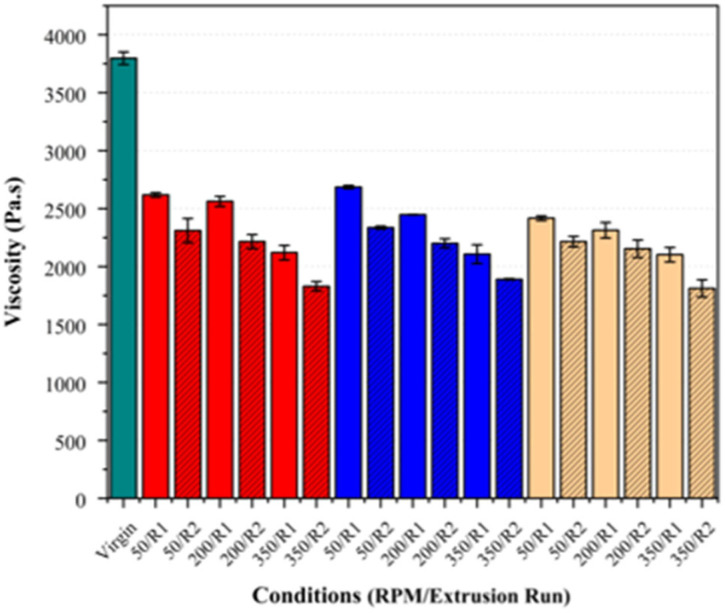
Zero-shear viscosity of PLA/basalt fibre composites at varying screw speeds (50, 200, and 350 RPM) and after different extrusion runs (R1, R2). Bar colours correspond to fibre loadings as defined in Figure 8.

Regarding the second extrusion run, the samples consistently exhibited an increase in their crossover frequency and a reduction in their zero-shear viscosity across all the fibre loadings and screw speeds (see Figure 12 and Figure 13). These changes were the most pronounced at higher screw speeds, supporting the inference that increased shear accelerates PLA degradation, particularly in the presence of basalt fibre.

### 3.4. Mechanical Properties

#### Tensile Testing

Tensile testing was performed to evaluate Young’s modulus (YM), the ultimate tensile strength (UTS), and the elongation at break, with the results presented in Table 5.

At 50 RPM, Young’s modulus exhibited increases with the fibre content of 5.43% (3478.79 MPa), 15.90% (3824.06 MPa), and 25.58% (4110.53 MPa) in the composites with 5%, 7.5%, and 10% fibre loadings, respectively, relative to that of the virgin PLA (3299.40 Mpa). This increase was consistent with the expected reinforcing effect of basalt fibres, attributed to their higher intrinsic stiffness [44,45]. Increasing the screw speed further to 200 and 350 RPM did not result in any notable changes. This was likely due to the lack of substantial differences in the fibre dispersion, as indicated by SEM.

**Figure 14:**
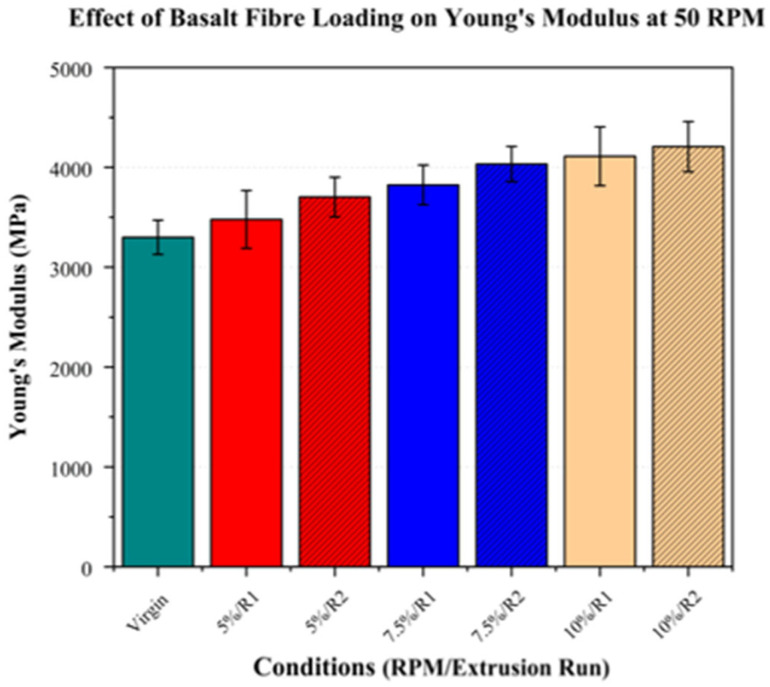
Young’s modulus of PLA/basalt fibre composites at 50 RPM for different fibre loadings (5%, 7.5%, and 10%) and extrusion runs (R1, R2). Virgin PLA is shown in green, with fibre loadings represented by red (5%), blue (7.5%), and beige (10%). Hatched bars indicate samples processed using second extrusion run (R2).

Following the second extrusion run, a trend of an increasing Young’s modulus was observed across all the fibre loadings, with increases of 12.23% (3702.99 MPa), 22.23% (4033.06 MPa), and 27.52% (4207.56 MPa) for the 5%, 7.5%, and 10% fibre loadings, respectively, compared to that of virgin PLA (3299.40 MPa). This improvement can be attributed to enhanced fibre dispersion, as evidenced through SEM imaging and ash content analysis. Additionally, SEM at 50× magnification showed a higher degree of fibre alignment in the tensile direction, which would also contribute to an enhanced modulus. As with the first extrusion run, increasing the screw speed to 200 RPM did not result in any notable improvements. However, at 350 RPM, a reduction was observed across all the fibre loadings. This decrease was likely due to high molecular weight loss, as indicated by the rheological analysis. The Young’s modulus values of the PLA/basalt fibre composites at 50 RPM across all the fibre loadings are presented in Figure 14.

Regarding the tensile strength, the highest values were observed at 50 RPM, with increases of 4.37% (72.25 MPa), 12.26% (77.21 MPa), and 15.95% (80.26 MPa) in composites with 5%, 7.5%, and 10% fibre loadings, respectively, compared to that of virgin PLA (69.22 MPa). These findings are consistent with those of Liou et al., who reported that lower screw speeds enhance mechanical properties by preserving the fibre length, which contributes to increased tensile strength [46].

With regard to the screw speed, as previously observed with Young’s modulus, increasing the screw speed from 50 RPM to 200 RPM did not result in a notable change. However, a notable reduction was observed at 350 RPM across all the fibre loadings, attributed to higher molecular weight degradation as a result of increased shear.

**Figure 15:**
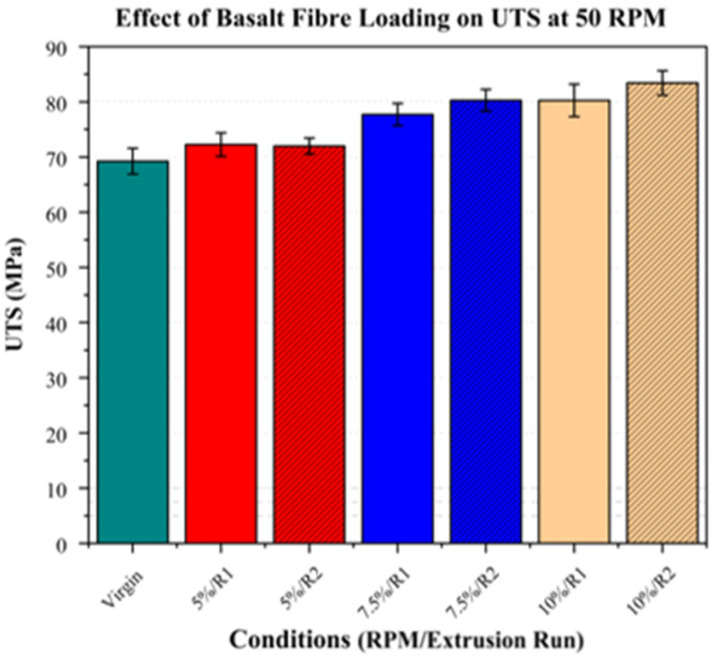
Ultimate tensile strength (UTS) of PLA/basalt fibre composites at 50 RPM for different fibre loadings (5%, 7.5%, and 10%) and extrusion runs (R1, R2). Virgin PLA is shown in green, with fibre loadings represented by red (5%), blue (7.5%), and beige (10%). Hatched bars indicate samples processed using second extrusion run (R2).

Following the second extrusion run, while the tensile strength at a 5% fibre loading remained largely unchanged, a trend of increased tensile strength was observed at 7.5% and 10% fibre loadings, reaching 80.26 MPa (15.05%) and 83.43 MPa (20.23%), respectively, compared to that of virgin PLA (69.22 MPa). These observed increases were attributed to the same factors responsible for the observed modulus improvements, i.e., enhanced fibre dispersion and a higher degree of fibre alignment in the tensile direction. As in the first extrusion run, increasing the screw speed beyond 50 RPM did not yield further improvements. The tensile strength of the PLA/basalt fibre composites at 50 RPM across all the fibre loadings is presented in Figure 15.

With respect to the elongation at break, a reduction was observed with an increasing basalt fibre loading across all the conditions. While no improvement in the elongation was expected due to the inherently low elongation of basalt fibres, the slightly lower values observed may be attributed to poor adhesion between the fibre and matrix [47].

## 4. Conclusions

The aim of this study was to investigate whether optimised processing conditions, specifically concerning the screw speed and a secondary extrusion run, could enhance the reinforcing effect of basalt fibre in PLA, thereby maximising its mechanical performance at low fibre loadings. Our findings indicate that lower screw speeds (50 RPM) are more favourable overall than higher speeds, as they maintained adequate fibre dispersion while minimising the molecular weight loss, ultimately leading to improved mechanical properties. As increasing the screw speed did not provide a clear benefit, lower screw speeds are recommended for more optimal processing in future research. Furthermore, implementing a second extrusion run proved effective, as it enhanced the fibre dispersion and orientation, ultimately contributing to greater strength and modulus values. As a result, PLA reinforced with basalt fibre under these conditions presents a promising alternative to virgin PLA for applications requiring greater strength and stiffness.

Nonetheless, several limitations were encountered that should be addressed in future research. For example, while 50 RPM was identified as the most effective screw speed, the impact of lower speeds should be evaluated. Further reductions in the screw speed could help minimise molecular weight degradation by reducing the shear stress, which may, in turn, contribute to additional improvements in the strength and modulus. However, lower speeds will increase the residence time and may also negatively impact the fibre dispersion and molecular weight degradation, introducing a trade-off that remains unclear. Consequently, future studies should determine the optimal screw speed that balances the residence time, fibre dispersion, and molecular weight retention.

A further limitation observed across all the samples was low fibre–matrix adhesion. Future research should explore alternative surface treatment strategies, such as plasma treatment or the use of surface coatings, to enhance adhesion, improving the mechanical performance in turn. Moreover, while the second extrusion run improved the fibre dispersion, it also led to notable changes in the rheological behaviour, suggesting a reduction in the molecular weight. This could adversely affect the composite’s long-term integrity, particularly under degradation conditions. Furthermore, the second extrusion run resulted in a further reduction in the fibre–matrix adhesion, likely due to excessive shear, which may have compromised the load transfer efficiency and overall mechanical integrity. Therefore, future studies should explore less aggressive secondary processing strategies, such as optimising the temperatures, lowering the screw speeds, or redesigning the screws to minimise the shear.

Additionally, the potential influence of the crystallinity was not assessed. Given that basalt fibres can act as nucleating agents, thereby promoting crystallisation, this will be investigated in future studies to evaluate its contribution to the changes in mechanical performance observed in this study. Finally, PLA is inherently brittle, and this limitation was further exacerbated by the addition of basalt fibre. This presents a critical challenge for stent applications, which require high ductility to withstand both crimping and expansion. Consequently, future studies should focus on strategies to enhance ductility while maintaining mechanical strength.

Overall, the findings in this study indicate that incorporating basalt fibre into PLA enhances its mechanical properties, suggesting that a thinner strut profile could be achieved compared to when using PLA alone. While increasing the basalt fibre content would likely provide further improvements, this study demonstrates that optimising the processing conditions, particularly by maximising the adhesion and dispersion and minimising the molecular weight loss, allows for enhancements at lower loadings, in turn reducing the risk of premature degradation due to a reduced PLA matrix proportion. These findings highlight the influence of the processing conditions on the mechanical performance of PLA/basalt fibre composites and provide a foundation for future research in this area.

## Figures and Tables

**Table 1:** Blend compositions for PLA/basalt fibre samples.

BF (wt.%)	PLA (g)	Basalt Fibre (g)
0	1000	0
5	950	50
7.5	925	75
10	900	100

**Table 2:** Samples selected for SEM analysis.

Basalt Fibre Loading (%)	Screw Speed (RPM)	Run	Purpose
0 (Virgin PLA)	200	R1	Control
0 (Virgin PLA)	200	R2	
5	200	R1	Effect of fibre loading
7.5	200	R1	
10	200	R1	
7.5	50	R1	Effect of screw speed
7.5	200	R1	
7.5	350	R1	
7.5	200	R1	Effect of extrusion run
7.5	200	R2	

**Table 3:** Ash content results for PLA/basalt fibre composites processed at varying screw speeds (50, 200, and 350 RPM) and after different extrusion runs (run 1 and run 2 donated as R1 and R2). Results represent average fibre content of three samples per condition and are reported with their standard deviation (σ).

Target Fibre Loading (%)	Conditions (RPM/Extrusion Run)	Actual Fibre Content (%)
5%	50/R1	4.10 ± 0.13
	50/R2	4.75 ± 0.03
	200/R1	4.36 ± 0.05
	200/R2	4.86 ± 0.04
	350/R1	4.41 ± 0.07
	350/R2	4.87 ± 0.01
7.50%	50/R1	6.48 ± 0.07
	50/R2	7.41 ± 0.03
	200/R1	7.08 ± 0.12
	200/R2	7.72 ± 0.05
	350/R1	7.11 ± 0.19
	350/R2	7.74 ± 0.05
10%	50/R1	8.71 ± 0.05
	50/R2	10.09 ± 0.07
	200/R1	10.12 ± 0.27
	200/R2	9.90 ± 0.06
	350/R1	10.15 ± 0.22
	350/R2	10.10 ± 0.02

**Table 4:** Rheological analysis of PLA/basalt fibre composites at varying fibre loadings (5%, 7.5%, and 10%) and screw speeds (50, 200, and 350 RPM) and after different extrusion runs (R1, R2).

Fibre Loading (%)	Conditions (RPM/Extrusion Run)	Crossover Point (rad/s)	Zero-Shear Viscosity (Pa·s)
Virgin	—	169.00 ± 0.71	3796.80 ± 53.60
5	50/R1	208.65 ± 5.66	2617.51 ± 19.30
	50/R2	278.55 ± 14.28	2309.74 ± 104.35
	200/R1	240.918 ± 0.90	2562.42 ± 43.60
	200/R2	284.56 ± 10.82	2215.52 ± 59.90
	350/R1	270.91 ± 6.40	2119.73 ± 62.76
	350/R2	305.06 ± 20.63	1829.53 ± 40.09
7.5	50/R1	237.62 ± 0.07	2685.14 ± 13.90
	50/R2	295.16 ± 12.61	1935.29 ± 13.66
	200/R1	255.20 ± 4.02	2446.86 ± 2.33
	200/R2	263.02 ± 12.32	2200.02 ± 38.60
	350/R1	286.35 ± 8.39	2106.46 ± 80.96
	350/R2	331.07 ± 3.19	1889.74 ± 5.59
10	50/R1	244.05 ± 2.76	2416.65 ± 19.80
	50/R2	267.31 ± 8.23	2215.33 ± 45.76
	200/R1	253.70 ± 4.57	2312.44 ± 66.90
	200/R2	270.849 ± 8.10	2153.46 ± 75.31
	350/R1	283.40 ± 8.57	2002.23 ± 62.62
	350/R2	322.99 ± 8.73	1811.29 ± 74.73

**Table 5:** Tensile properties with standard deviations of replicates (*n* = 5) of PLA/basalt fibre composites at varying fibre loadings and screw speeds and after different extrusion runs.

**Fibre Loading (%)**	**Conditions (RPM/Run)**	**YM (MPa)**	**UTS (MPa)**	**Elongation (%)**
-	Virgin PLA	3299.40 ± 172	69.22 ± 2.34	3.89 ± 0.05
5	50/R1	3478.79 ± 89	72.25 ± 2.12	2.87 ± 0.06
	200/R1	3605.63 ± 115	74.78 ± 2.87	2.86 ± 0.05
	350/R1	3527.81 ± 106	71.26 ± 1.98	3.09 ± 0.07
	50/R2	3702.99 ± 98	71.97 ± 1.45	2.88 ± 0.06
	200/R2	3721.33 ± 79	67.31 ± 2.63	2.94 ± 0.06
	350/R2	3689.69 ± 111	66.3 ± 1.79	2.73 ± 0.08
7.5	50/R1	3824.06 ± 99	77.71 ± 2.01	2.84 ± 0.05
	200/R1	3820.19 ± 78	77.35 ± 2.56	2.85 ± 0.06
	350/R1	3881.81 ± 11	73.11 ± 3.22	2.76 ± 0.05
	50/R2	4033.06 ± 76	80.28 ± 1.97	2.86 ± 0.04
	200/R2	4038.9 ± 85	80.79 ± 3.18	2.9 ± 0.05
	350/R2	3801.5 ± 84	74.78 ± 2.75	2.85 ± 0.06
10	50/R1	4110.53 ± 94	80.26 ± 2.94	2.68 ± 0.06
	200/R1	4151.81 ± 85	76.35 ± 1.88	2.56 ± 0.07
	350/R1	4169.88 ± 112	75.82 ± 2.43	2.75 ± 0.05
	50/R2	4207.56 ± 121	83.43 ± 2.21	2.92 ± 0.05
	200/R2	4137.35 ± 102	76.21 ± 1.76	2.85 ± 0.06
	350/R2	4065.44 ± 106	77.86 ± 2.89	2.81 ± 0.06

## Data Availability

The original contributions presented in this study are included in the article. Further inquiries can be directed to the corresponding authors.
